# Ethnomedicinal study of plants used for human ailments in Ankober District, North Shewa Zone, Amhara Region, Ethiopia

**DOI:** 10.1186/1746-4269-9-63

**Published:** 2013-08-28

**Authors:** Ermias Lulekal, Zemede Asfaw, Ensermu Kelbessa, Patrick Van Damme

**Affiliations:** 1 Laboratory for Tropical and Subtropical Agriculture and Ethnobotany, Department of Plant Production, Faculty of Bio-Science Engineering, Ghent University, Coupure links 653, 9000 Gent, Belgium; 2 Department of Plant Biology and Biodiversity Management, College of Natural sciences, Addis Ababa University, P.O. Box 3434, Addis Ababa, Ethiopia; 3 Department of Crop Science and Agroforestry, Institute of Tropics and Subtropics, Czech University of Life Sciences Prague, Kamycka 129, 165 21 Prague 6-Suchdol, Czech Republic

**Keywords:** Ethnomedicine, Fidelity level, Informants consensus factor, Indigenous knowledge, Medicinal plants, Traditional healers

## Abstract

**Background:**

Ankober District has long been inhabited by people who have a long tradition of using medicinal plants to treat human ailments. Overexploitation of medicinal plants coupled with an ever-increasing population growth, deforestation and agricultural land expansion threatens plants in the area. Hence, this study aimed at documenting and analyzing the plant-based ethnomedicinal knowledge of the people in order to preserve the dwindling indigenous knowledge.

**Methods:**

Ethnobotanical data were collected using semi-structured interviews, focus group discussions, participant observation and walk-in-the-woods. Quantitative approaches were used to determine Informant Consensus Factor (ICF) and Fidelity level (FL) values. Statistical tests were used to compare the indigenous knowledge on medicinal plants among different informant categories.

**Results:**

A total of 135 medicinal plant species belonging to 128 genera and 71 botanical families were reported to treat human diseases in the District. Families Asteraceae (12 species, 9%) and Fabaceae (10, 7.4%) were found to be best represented in the area. About 44% of preparations were reported to be obtained from roots. Significant difference (P < 0.05) was observed on the mean number of medicinal plants reported by groups of respondents compared within age, literacy level and experience parameters. Highest ICF values were recorded for gastro-intestinal & parasitic and dermatological disease categories (0.70 each) indicating best agreement among informants knowledge on medicinal plants used to treat aliments in these categories. Highest fidelity level values were recorded for *Zehneria scabra* (95%) and *Hagenia abyssinica* (93.75%) showing conformity of knowledge on species of best healing potential. *Podocarpus falcatus* was ranked first in a direct matrix ranking exercise of multipurpose medicinal plants*.* The output of preference ranking exercise indicated that *Olea europaea* subsp. *cuspidata* was the most preferred species to treat atopic eczema.

**Conclusion:**

The study revealed that Ankober District is rich in medicinal plant diversity and associated indigenous knowledge. However, anthropogenic factors coupled with acculturation and very poor conservation efforts threaten medicinal plant survival in the area. Promoting a complementary *in situ* and *ex situ* conservation strategy for medicinal plants of the District is highly recommended.

## Introduction

Knowledge on plant use is the result of many years of man’s interaction and selection on the most desirable, the most vigorous and the most successful plant present in the immediate environment at a given time [1]. The need for well-being of a society is an ultimate driver of millennia old interaction and selection of most successful medicinal plants and development of indigenous knowledge associated with utilization of curative plants. According to [2], traditional knowledge on plant use will be lost in the absence of continuous cultural interaction. Demographic, economic, socio-political, ecological, religious and cultural entities existing in a community are key drivers of traditional knowledge in a given community [3]. Various ethnomedicinal investigations also show that traditional knowledge on medicinal plants varies depending on different factors including gender, age and occupation [4-6].

Traditional plant remedies are the most important source of therapeutics for nearly 80% of the developing world population [7]. The same is true in Ethiopia where medicinal plants play a significant role in supporting the country’s primary healthcare system [8,9]. About 95% of traditional medicine preparations in Ethiopia are mentioned to be of plant origin [10]. The deep-rooted culture of using medicinal plants in the country led the people to be acquainted with knowledge of medicinal properties of many plants used to treat human and livestock ailments [11]. Although, ancient medico-religious pharmacopeias of Ethiopian medicinal plants written on parchments in the classical Geez language (now the working language only in the Ethiopian Orthodox Tewahdo Church) have documented part of the indigenous knowledge on utilization of medicinal plants most of the documents are lost due to damage, theft and illegal selling to foreign plant collectors [12]. The country’s plant lore has also received a lot of attention from many foreign travellers as evidenced by [13-16] who documented Ethiopian medicinal plants originated from the then medico-religious writings.

Although medicinal plants play a significant role in supporting the primary healthcare in Ethiopia, only a limited attempt has been done to scientifically explore, document and promote the widely used medicinal plants and associated knowledge dynamics in the country. Scientific investigation of millennia old community knowledge on plant use is crucial to define cultural identities of a particular community and understand links to their history, land and plant use practices and traditional environmental philosophy [3,17]. In addition, it helps to design people centred natural resource management practice which is important for biodiversity conservation [18,19]. Investigating traditional knowledge on medicinal plant use has also been used a basis for the discovery of new lead compounds that are used for the development of modern drugs [20].

The knowledge on traditional medicinal plants of Ethiopia which was developed for millennia is now subjected to loss since it has mainly been stored in the memories of elderly peoples and handed down mostly by word of mouth for successive generations [21]. Moreover, deforestation, overexploitation, overgrazing, habitat loss and degradation, agricultural land expansion and acculturation continuously threat Ethiopian traditional medicinal plants and linked knowledge [22]. Hence, it is a timely endeavour to investigate, document and analyze traditional knowledge on medicinal plants and associated knowledge drivers, so that sound medicinal plant utilization and management practices can be maintained. Moreover, it provides the opportunity for recognition, promotion, management and protection of indigenous knowledge of a community on medicinal plants as vital part of a nation’s heritage, beside calling policy makers, natural resource managers, stake holders and cultural practitioners for conservation actions.

Recent publications on Ethiopian medicinal plants including those by [21,23-35] attempt to address traditional uses of medicinal plants and associated knowledge in some cultural groups but are insignificant when compared to the 85 diverse ethnolinguistic communities in the country, most of them largely unexplored. Hence, the present research aims to fill this gap by documenting the wealth of indigenous knowledge and understanding the corresponding drivers of this knowledge related to utilization, management and conservation of medicinal plants used to treat human ailments in Ankober District, north Shewa Zone, Ethiopia which has never been explored for its ethnomedicinal wealth. The study also aims to identify and document marketable medicinal plants of the District to identify the money-making potential of medicinal plants in the area. In addition, the study aims to select candidate medicinal plant species of high informants’ consensus value for phytochemical and pharmacological analyses in our subsequent studies.

## Materials and methods

### Study area and ethnographic background

**Figure 1:**
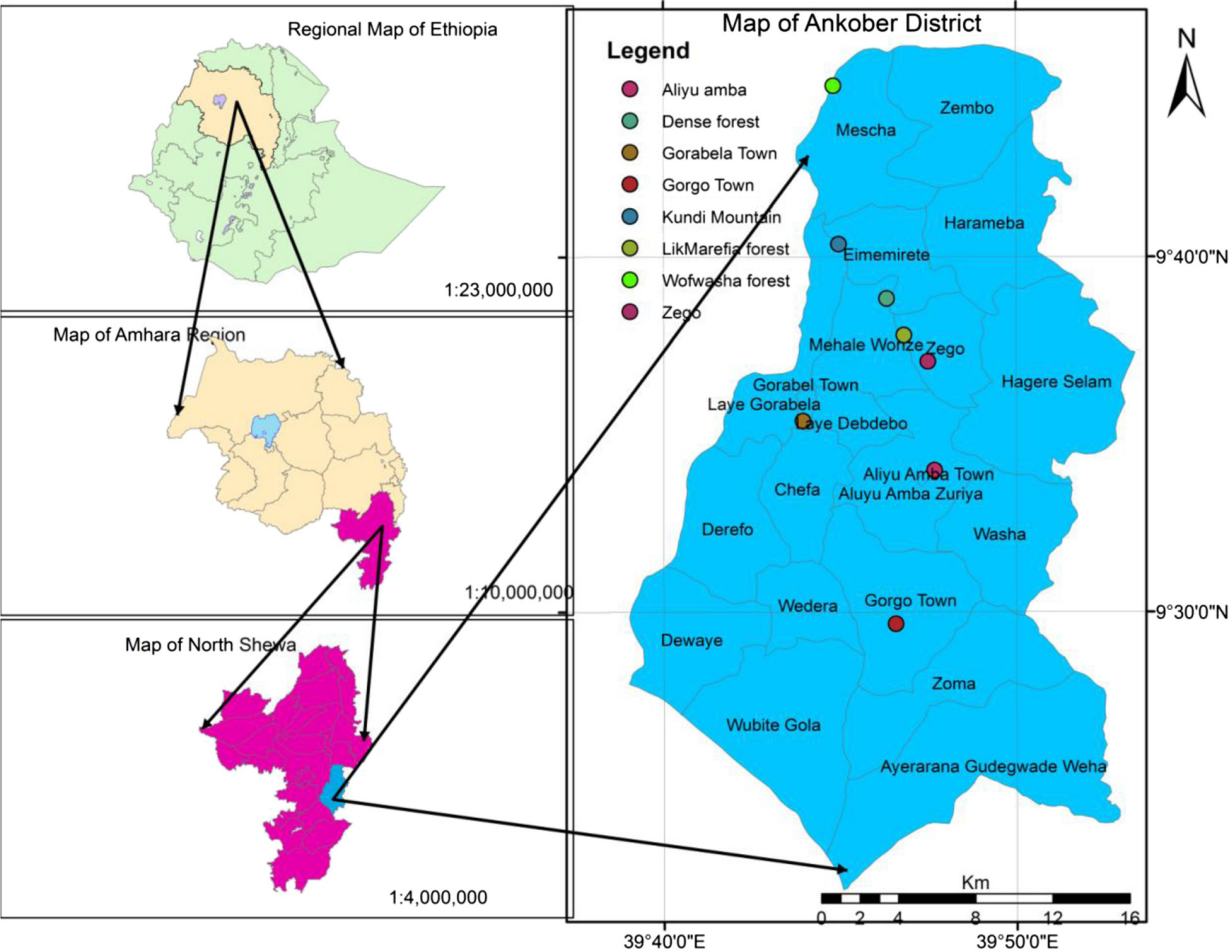
Map of the study area.

This study was conducted in Ankober Distrcit, located in north Shewa Zone of Amhara National Regional State in north-central Ethiopia (Figure 1). The District is perched on the eastern escarpment of the Ethiopian highlands and situated 172 km north of Addis Ababa, the Ethiopian capital, and 42 km to the east of Debre Berhan town (the north Shewa Zone capital) at 9° 22’ - 9° 45’ N and 039° 40’ - 039° 53’ E. Ankober District is bordered in the north by Tarmaber, south by Asagirt and west by Basonaworana Districts of Amhara Region. The eastern part share its border with Gachine Special District of the Afar Region [36]. The elevation of Ankober District ranges from 1300 m asl near Addis Alem area to 3700 m asl at Kundi mountain. The main administrative centre of the District is located at Gorabela/Ankober town that has historical significance as it has been the seat of the Ethiopian emperors from 1270 for centuries [36].

The indigenous people inhabiting the area belong to the Amhara ethnic group. They speak Amharic language, the national language of Ethiopia. The District has a total population of 83,260 (42,180 men and 41,080 women) of whom only 6,272 (7.5%) are urban inhabitants [37]. Ankober has a population density of 113.72 individuals/km^2^, which is slightly less than the north Shewa Zone average of 115.3 persons/km^2^[37]. About 92.52% of the people in Ankober belong to the Ethiopian Orthodox Tewahdo Christianity and 7.41% are Muslims.

The District has long been inhabited by people who have a long tradition of using plants and much of the land has now been converted in to cropland including the very steep slopes where cultivation is being undertaken by terracing the cliffs. Use of plants by people continued over generations to the present time and circumstantial evidence suggests that the main sources of plants for traditional medicine are the remaining forest patches, cultivated land and field margins. The District includes Wof Washa, Dense and Likmarefia natural forests. It is characterised by cold temperatures for most of the year. Its annual rainfall ranges from 1000 to 1400 mm [37]. Forests in Ankober District are amongst the richest biodiversity areas in the highlands of Ethiopia housing economically important tree species including *Hagenia abyssinica* (Bruce) J.F. Gmel., *Olea europaea* L. subsp. *cuspidata* (Wall. ex G. Don), *Juniperus procera* L*.*, *Podocarpus falcatus* (Thunb.) Mirb. and *Nuxia congesta* R. Br. ex Fresen [38]. They are also home to very diverse wildlife and bird species including the red-listed endemic bird species *Serinus ankoberensis*[39].

### Informant selection

The ethnomedicinal survey involved a total of 352 informants (235 male and 117 female) from all 22 kebeles (lowest administrative units in Ethiopia) of Ankober District. Systematic random and purposive sampling methods were employed to select representative general informants and knowledgeable traditional herbalists following the methods described by [26,33]. Informants’ ages ranged from 20–89 years old (122 were between 20–39 whereas 230 were ≥ 40 years old). Nominations on traditional herbalists to participate as key respondents were collected from elderly people in the study kebeles and used to identify 88 key informants (68 men and 20 women) among the inhabitants, whereas general informants were sampled during random visits made to houses in the study kebeles. Informed consent was obtained before the start of interviews from each general informant and traditional healer who participated in this study.

### Data collection

The ethnobotanical survey was carried out in five different field trips made between 25 June 2009 and 7 May 2011. Data were collected in different seasons of multiple years with the objective of addressing all kebeles in the District and collecting different plant specimens during flowering seasons [26,33]. Market survey and checking reliability of informants’ medicinal plant use information were conducted between 29 December 2012–9 February 2013.

Ethnobotanical data were collected in very close interaction with informants using semi-structured interviews, focus group discussions, participant observation and walk-in-the-woods [40,41]. Interviews were conducted in Amharic language and run independently for each informant. Interviews addressed issues regarding the name, age, sex, level of education, occupation, religion, and ethnicity of informants. Moreover, informants were asked about local names of medicinal plants used, ailments treated, habitat of the species, distance to gathering sites, seasonality of species, marketability of species, degree of management (wild/cultivated), abundance, parts used, condition of plant part used (fresh/dried), other ingredients or additives (if any), methods of remedy preparation, remedy preservation (storage), dosage prescriptions, routes of remedy administration, noticeable adverse effects of remedies, use of antidotes for adverse effects, taboos/beliefs related to collection and use of plants, source of knowledge, method of indigenous knowledge transfer, number of years of service as traditional healer, income earned per patient treated, other uses of medicinal plant species, existing threats and traditional conservation practices (if any) following the methods from [40-43]. All semi-structured interviews were followed by independent walk-in-the-woods which gave an opportunity for more discussion with the informant and the practical identification of traditionally used medicinal plants in the natural environment. This method was combined with the participant observation practice through which reliable information was obtained on the how of collection and preparation of specific remedial parts [40,42]. In addition, focus group discussions were also designed so as to gain further information on medicinal plants knowledge of the community and prove the reliability of the data collected through semi-structured interviews [41].

Data on use diversity of multipurpose medicinal plants were evaluated by a direct matrix ranking exercises as described in Cotton [40] that involved fifteen (ten men and five women) key informants. Participants for this exercise were selected based on their long years of experience as traditional herbal medicine practitioners in the District as described in Yineger *et al.*[33]. The same key informants also participated in a preference ranking exercise in the manner recommended by Martin [41] to identify the most preferred species for treating the most commonly reported dermatological disease in the area.

A market survey of medicinal plants of the District was conducted at six major markets i.e., namely, Gorebella, Aliyuamba, Gorgo, Haramba, Derefo, and Zego. Availability, price and unit of measurement of each marketable medicinal plant was documented and analysed so as to identify extent of use and income generating potential of the respective medicinal plants.

Interviews and discussions were all followed by voucher specimen collection that was held with the help of traditional healers and local field assistants. Specimens were air-dried, numbered, labelled, pressed, heater-dried, deep-freezed, identified and deposited at the National Herbarium (ETH) in Addis Ababa University. Identification of specimens was performed both in the field and later at ETH using taxonomic keys and floras [44-51] and by comparison with authenticated herbarium specimens.

### Data analysis

Data on informants’ backgrounds and medicinal plants used in Ankober were entered in an Excel spreadsheet software (Microsoft corporation, 2007) and organised for statistical analysis. Traditional knowledge dynamics on use of medicinal plants by men and women, young to middle aged (23–39 years) and elderly (40–89 years); literate (completed at least primary education) and illiterate; knowledgeable (key) and local (encountered randomly) informants as well as those living near health centres (≤ 5 km from health centres) and far (> 5 km distance from health centres) was compared using t-test and one way ANOVA at 95% confidence level between means following [33] using KyPlot 5.0 software. Descriptive statistics were also applied to identify the number and percentage of species, genera and families of medicinal plants used, their growth forms, proportions of parts harvested, modes of remedy preparation and routes of administration in the same manner as described by [26]. Values or scores given by key informants on use-preference and/or use-diversity of medicinal plants were added and ranked to get the output of preference ranking and direct matrix ranking exercises, respectively, following [41,42].

Informant consensus factor (ICF) was computed after the reported traditional remedies and corresponding diseases were grouped into 12 categories. ICF was obtained by computing number of use citations in each disease category (n_ur_) minus the number of times a species used (n_t_), divided by the number of use citations in each category minus one [52].

ICF = n_ur-_ n_t/_ n_ur-1_

The relative healing potential of each reported medicinal plant used against human ailments was evaluated using the fidelity level (FL) index [42] given by FL = Ip/Iu × 100, where Ip is the number of informants who independently cited the importance of a species for treating a particular disease and Iu the total number of informants who reported the plant for any given disease.

## Results

### Diversity of reported medicinal plants

A total of 135 medicinal plant species belonging to 128 genera and 71 botanical families consisting of 68 angiosperms, 2 gymnosperms and 1 fern were reported to be used for treating human ailments in Ankober District (Additional file 1). The family Asteraceae was represented by the highest number of species (12 species, 9%) followed by Fabaceae (10, 7.4%), Solanaceae (7, 5.2%), Lamiaceae (6, 4%), Cucurbitaceae, Ranunculaceae and Rosaceae (4 species each, 3%). Eight of the reported families i.e., Acanthaceae, Asclepiadaceae, Celasteraceae, Myrsinaceae, Oleaceae, Rubiaceae, Rutaceae and Euphorbiaceae were represented by three species each, whereas families Amaranthaceae, Apiaceae, Apocynaceae, Boraginaceae, Polygonaceae, Sapindaceae, Scrophulariaceae and Urticaceae were represented by 2 species each. Each of the remaining 48 families had single species representation. Thus, 32% of families were represented by more than one medicinal plant species. Identified growth forms of medicinal plants indicated that herbs (51 species; 38%) were dominant than shrubs (43; 32%) or trees (32; 24%) and climbers (9; 7%). About 5% (7 species) of medicinal plants of Ankober are found endemic to Ethiopia (Additional file 1).

### Indigenous knowledge of the community

Although more number of medicinal plants were reported by men than those reported by women, the difference was not significant (P >0.05) when the average number of medicinal plants reported by each group was compared. However, there was a significant difference (P < 0.05) in the number of medicinal plants reported by senior members of the community (40–80 years old) and young to middle aged members (20–39 years old); key informants and general informants, illiterate and literate informants (Table 1). More number of medicinal plants were reported by elderly (≥ 40 years old) and key informants than young and general informants. There was no significant difference observed in the number of medicinal plants listed by informants living around (≤ 5 km) health centres and those living relatively far away (>5 km) from the health centres.

**Table 1:** Statistical test of significance, t-test, on average number of reported medicinal plants among different informant groups in Ankober District

**Parameters**	**Informant groups**	**N**	**Average ± SD**	t -value**	**p –value**
Gender	Male	235	4.23 ± 0.13	1.61	0.1075
	Female	117	3.85 ± 0.19		
Age	Young members	122	2.59 ±0.08	−11.65	0.0001*
	Senior members	230	4.90 ± 0.13		
Literacy level	Illiterate	85	4.73 ± 0.11	−11.90	0.0001*
	Literate	267	2.12 ± 0.10		
Distance from health centres	Proximity to health centre	80	4.22 ± 0.22	0.57	0.5681
	Far away from health centre	272	4.07 ±0.12		
Experience (Informant category)	Key/knowledgeable	88	6.94 ± 0.16	23.88	0.0001*
	General informant	264	3.16 ± 0.07		

*Significant difference (p < 0.05); ** t(0.05) (two tailed), df = 350, N = number of respondents.

### Disease types and treatment methods

About 69 disease types were reported in Ankober District for which traditional healers were visited at least once (Additional file 1). Constipation, diarrhoea and taeniasis were the most commonly reported health problems under the gastro-intestinal disease category, whereas atopic eczema was most frequently reported under the dermatological disease group.

Visual inspection and interview were the commonly reported diagnosis methods prior to any herbal medicine prescription in the society. Depending on types of reported ailments, traditional healers diagnose patients with an interview for symptoms followed by visual inspection of eyes, skin colour, tongue, throat, status of sores, bleeding, infections and sensing body temperature of their patients with their bare hands. Patients with skin infections were reported to be treated by rubbing and pasting herbal preparations whereas those with sores were treated by chewing the part of the medicinal plant and spitting the juice on the sore. For internal ailments, herbal preparations were mainly prescribed to be administered orally whereas for a general malaise steam bath and vapour inhalation were commonly reported.

### Plant parts used for remedy preparation

**Figure 2:**
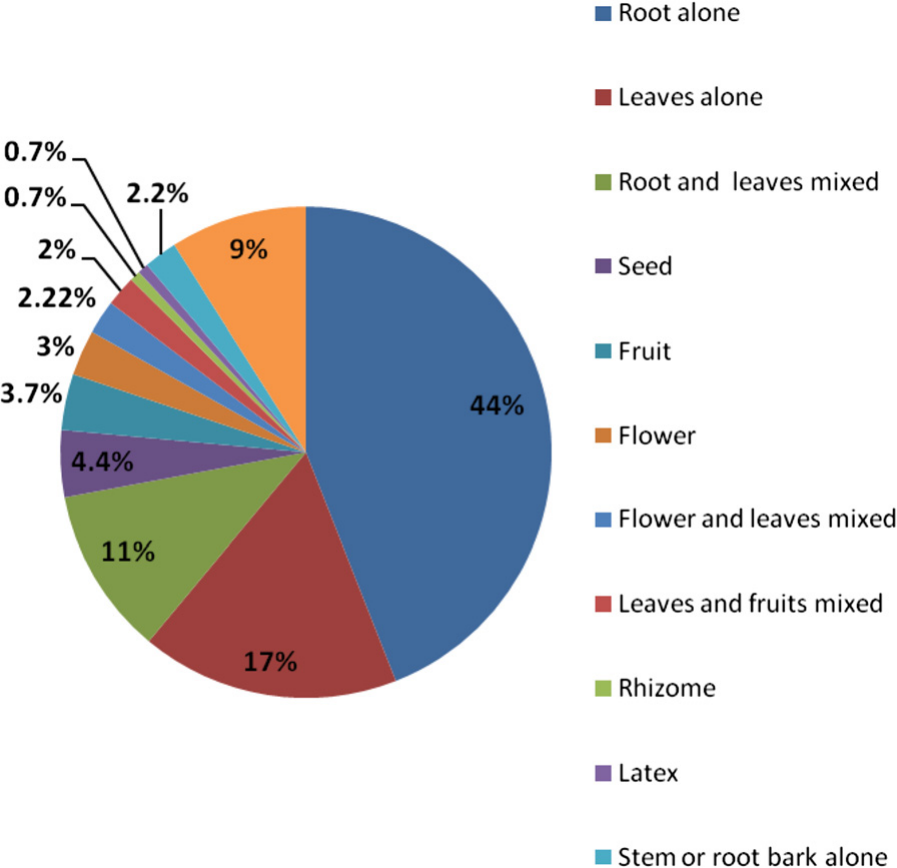
Plant parts used for remedy preparation in Ankober District.

Despite mentioning different plant parts used for remedy preparation, the majority (44%) of preparations were from root parts alone followed by leaves (17%) alone and mixtures of roots and leaves (11%) (Figure 2). Plants in which roots (82 species, 61%) and leaves (48 species, 36%) are utilized as medicine either as sole or mixed with other plant parts were most frequent in the medicinal flora of the District. Freshly harvested plant parts were the dominant ones (69.9%) used in remedy preparation whereas dried parts were used least (2.73%); the remaining 27.4% of remedies were reported to be prepared both from dried or fresh parts of medicinal plant species.

### Modes of remedy preparation and application

**Figure 3:**
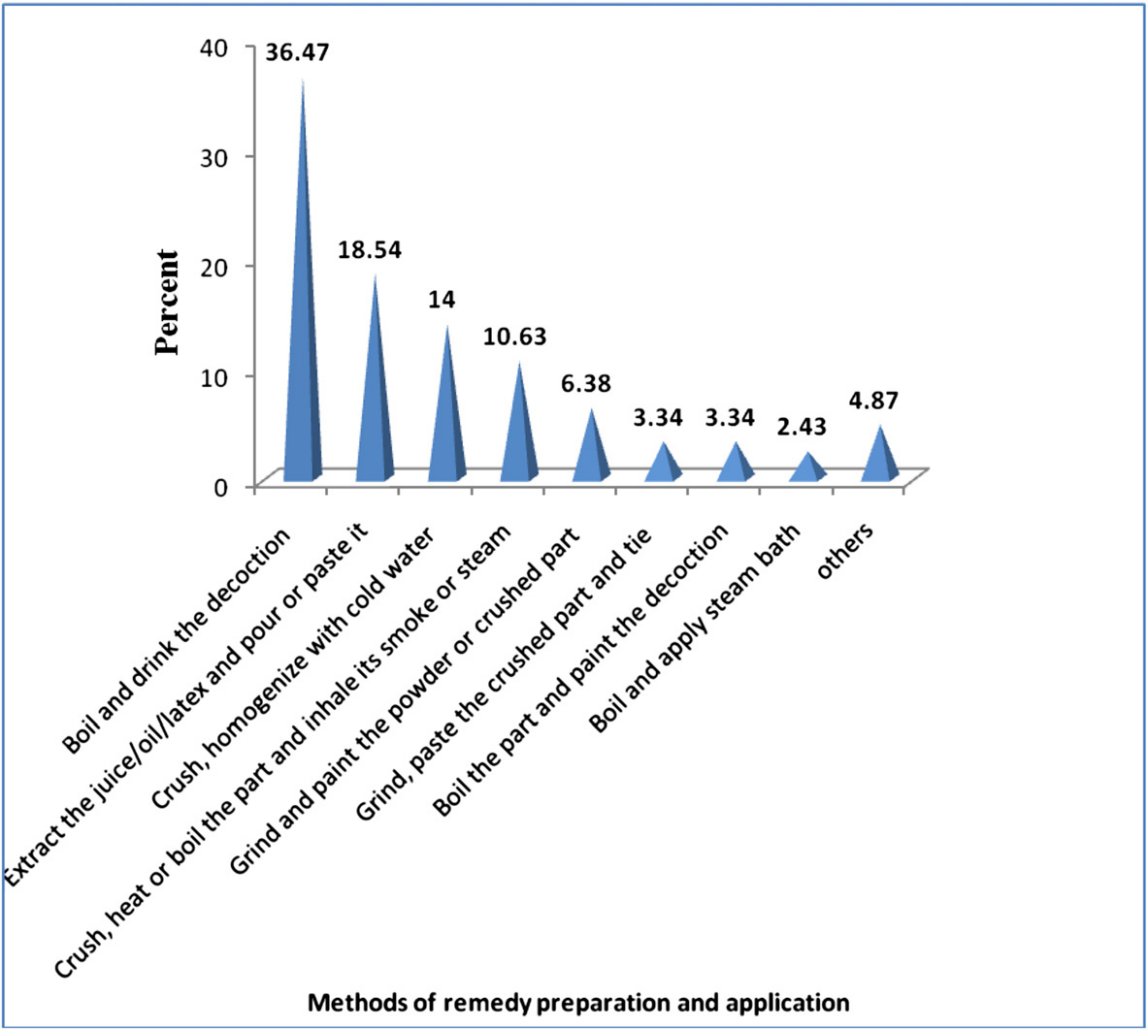
Modes of remedy preparation and application in Ankober District.

Traditional healers in the study area reported that they follow various ways of remedy preparation and this depends, according to their explanations, on the type of ailment. The major modes of remedy preparation list were decoction (36.47%); extracting juice, oil or latex from the plant (18.54%) and pounding and homogenizing plant parts (14%) (Figure 3).

### Routes of administration

Medicinal plant preparations were administered through different routes. Oral application (181 preparations, 55.01%) was the best represented and most commonly used route of administration followed by topical or dermal application (81 preparations, 25%). The remaining remedies were reported to be administered through nasal (42 preparations, 12.76%); auricular (12 preparations, 3.64%); anal (8 preparations, 2.43%) or optical (5 preparations, 1.51%) routes depending on the type of ailment reported by the respective patients.

### Dosages and antidotes

Most medicinal plants prescribed and given to patients are applied without any standardised doses. However, approximate dosages (although no fixed standards) were reported to be determined based on age, sex and physical appearance of patients visiting local healers. Some medicinal plant preparations were mentioned to be measured in small cups locally called YEBUNA SINI referring to traditional cups used for drinking coffee or plastic jugs, while others were measured as handful, spoonful or size of a finger. Coffee, milk, honey, yoghurt, butter and dissolved powder of roasted barley, locally known as BESSO*,* were commonly reported antidotes for herbal preparations with adverse side effects.

### Marketability of medicinal plants

Out of the 25 (19%) species reported as marketable, only five species i.e., *Echinops kebericho* Mesfin*, Embelia schimperi* Vatke, *Hagenia abyssinica* (Bruce) J.F. Gmel., *Withania somnifera* (L.) Dun. in DC. and *Silene macrosolen* A. Rich. were found on markets being sold and purchased entirely for the purposes of their medicinal applications. The remaining reportedly marketable medicinal plants were mainly sold for their non-medicinal uses but occasionally applied as medicine when the need arises. The average price of a cup (YEBUNA SINI) of *Embelia schimperi* fruits at Aliuamba, Derefo, Gorebella, Gorgo, Haramba and Zego local markets was 5.50 Eth Birr (0.3 USD), whereas for a bunch (**≈**250-325 gm) of the root material of *Echinops kebericho* and *Silene macrosolen* it was 4 Birr (0.21 USD); and the price was 3.50 Birr (0.2 USD) for a similar bunch of *Withania somnifera* roots. A jug of *Hagenia abyssinica* inflorescence was sold for 4 Birr (0.21 USD).

### Efficacy of medicinal plants

About twelve disease categories were identified from the total of 74 various human ailments reported in the District. Amongst these, the categories with the highest ICF values were gastro-intestinal & parasitic and dermatological diseases (0.70 each) which were followed by respiratory (0.65); and oral, dental and pharyngeal diseases (0.62) (Table 2). Highest plant use citation (19.75%) was found for gastrointestinal and parasitic diseases followed by dermatological diseases (12.46%).

**Table 2:** ICF values of traditional medicinal plants for treating human ailments in Ankober

**No**	**Disease category**	**Species**	**% all species**	**Use citations**	**% all use citations**	**ICF**
1	Gastro-intestinal and parasitic	20	14.81	65	19.75	0.70
2	Dermatological	13	9.62	41	12.46	0.70
3	Respiratory	7	5.18	18	5.47	0.65
4	Oral, dental and pharyngeal	6	4.44	14	4.25	0.62
5	Sensorial	8	5.92	17	5.16	0.56
6	Urogenital and venereal	15	11.11	30	9.11	0.52
7	Febrile	11	8.14	21	6.38	0.5
8	External injuries, bleeding and snake bite	13	9.62	19	5.77	0.33
9	Musculoskeletal and nervous system	21	15.55	28	8.51	0.26
10	Blood and lymphatic system	18	13.33	24	7.29	0.26
11	Evil spirit	21	15.55	22	6.68	0.05
12	Others	29	21.48	31	9.42	0.06

### Relative healing potential of medicinal plants

Highest fidelity level (95%) was recorded for *Zehneria scabra* (Linn.f.) Sond. followed by *Hagenia abyssinica* (Bruce) J.F. Gmel. (93.75%), *Ocimum lamiifolium* Hochst. (93.33%) and *Thalyctrum rhynchocarpum* Dill. & Rich. (91.6%) (Table 3). The recorded highest fidelity level values of *Zehneria scabra* and *Ocimum lamiifolium* were both obtained under the febrile therapeutic category. In contrast, the highest fidelity level values of *Hagenia abyssinica* and *Thalictrum rhynchocarpum* were both found from gastro-intestinal and parasitic disease categories.

**Table 3:** Fidelity Level value of medicinal plants commonly reported against a given ailment category

**No**	**Medicinal plant**	**Therapeutic category**	**Ip**	**Iu**	**FL value (%)**
1	*Zehneria scabra* (Linn.f.) Sond.	Febrile	19	20	95
2	*Hagenia abyssinica* (Bruce) J.F. Gmel.	Gastro-intestinal and parasitic	15	16	93.75
3	*Ocimum lamiifolium* Hochst.	Febrile	14	15	93.33
4	*Thalictrum rhynchocarpum* Dill. & Rich.	Gastro-intestinal and parasitic	22	24	91.6
5	*Echinops kebericho* Mesfin	Evil spirit	17	19	89.47
6	*Croton macrostachyus* Del.	Dermatological	23	26	88.46
7	*Embelia schimperi* Vatke	Gastro-intestinal and parasitic	14	16	87.5
8	*Cyatula cylinderica* Moq.	External injuries, bleeding and snake bite	11	13	84.6
9	*Jasminum abyssinicum* Hochst.	Gastro-intestinal and parasitic	10	13	76.92
10	*Olea europaea* L. subsp. *cuspidata* (Wall. ex G. Don)	Dermatological	15	18	83.33
11	*Asparagus africanus* Lam.	Urogenital and venereal	7	10	70

### Use diversity of medicinal plants

The output of the direct matrix ranking (DMR) exercise on ten multipurpose medicinal plants enabled to identify which of the multipurpose plants is most under pressure in the area and the corresponding factors that threaten the plant. Accordingly, *Podocarpus falcatus* was ranked first (most threatened) followed by *Olea europaea* subsp. *cuspidata* and *Ekebergia capensis* (Table 4). Results also indicated that those multipurpose medicinal plant species are currently exploited more for construction, firewood and lumbering purposes than for their medicinal role.

**Table 4:** Average DMR score of fifteen key informants for ten medicinal plants species with additional uses besides medicinal value

**Use diversity**	***B.abyssinica***	***C.africana***	***C.macrostachyus***	***D. torrida***	***E.capensis***	***E.globulus***	***J.procera***	***Olea europaea *subsp. *cuspidata***	***P.falcatus***	***P.africana***	**Total**	**Rank**
Agricultural tool	3	1	2	4	4	2	1	3	5	3	**28**	**5**
Construction	2	4	2	4	4	5	5	5	5	5	**41**	**1**
Lumbering	2	2	2	4	5	1	4	4	5	5	**34**	**3**
Firewood	4	3	4	5	4	5	4	5	3	3	**40**	**2**
Charcoal	2	3	1	3	3	0	3	3	4	1	**23**	**6**
Medicine	4	2	5	2	3	5	3	4	3	2	**33**	**4**
**Total**	**17**	**15**	**16**	**22**	**23**	**18**	**20**	**24**	**25**	**19**		
**Rank**	**8**	**10**	**9**	**4**	**3**	**7**	**5**	**2**	**1**	**6**		

Based on use criteria (5 = best; 4 = very good; 3 = good;2 = less used; 1 = least used and 0 = no value).

### Preference ranking

A preference ranking exercise with 15 key informants on medicinal plants that were reported to be used against atopic eczema, the most frequently reported disease in the dermatological disease category, showed that *Olea europaea* subsp. *cuspidata, Allium sativum* and *Datura stramonium* were the most preferred species to treat the reported disease (Table 5).

**Table 5:** Preference ranking of medicinal plants reported for treating atopic eczema

**Medicinal plants for atopic eczema**	**Informants labelled A to O**																
	**A**	**B**	**C**	**D**	**E**	**F**	**G**	**H**	**I**	**J**	**K**	**L**	**M**	**N**	**O**	**Total score**	**Rank**
*Allium sativum* L.	6	5	6	7	4	4	5	6	7	6	4	5	4	5	7	81	**2**
*Asparagus africanus* Lam.	1	2	3	2	1	3	1	2	1	7	3	1	1	7	1	36	**6**
*Clematis hirsuta* Perr. & Guill.	3	4	2	1	2	1	3	3	2	1	1	2	3	2	2	32	**7**
*Croton macrostachyus* Del.	4	3	4	5	7	5	4	4	3	4	7	3	5	6	3	67	**4**
*Datura stramonium* L.	5	7	5	3	6	7	6	5	6	3	5	4	6	3	5	76	**3**
*Olea europaea* L. subsp. *cuspidata* (Wall. ex G. Don)	7	6	7	6	5	6	7	7	5	5	6	6	7	4	6	90	**1**
*Solanum anguivi* Lam*.*	2	1	1	4	3	2	2	1	4	2	2	7	2	1	4	38	**5**

**N:B**-Scores in the table indicate ranks given to medicinal plants based on their efficacy. Highest number (7) given for the medicinal plant which informants thought most effective in treating atopic eczema and the lowest number (1) for the least effective plant.

### Indigenous knowledge transfer

The major way of indigenous knowledge transfer on types of medicinal plants, traditional concepts of illness and methods of diagnosis among traditional healers of Ankober District was by word of mouth to a family member, specially of an elder son. It was also found that there is maximum secrecy in passing the knowledge within the family circle. None of the participants had written documents on traditional medicine whereas all healers reported that they received the knowledge from their parents or grandparents orally. The way they share their indigenous knowledge to their children was also found to be similar.

### Conservation practices

Although traditional practitioners and local communities of Ankober District mainly depend on the wild environment for collecting medicinal plants, the effort to conserve and sustainably utilize resources was found frail. Despite harvesting majority of medicinal plants (99 species, 73%) from the wild environment alone no attempt of *in situ* conservation was observed to save fast eroding medicinal plants of the District. About 15% (20 species) of medicinal plants of the District are available from cultivation (Additional file 1) whereas the remaining 12% (16 species) were reported to be harvested both from home gardens and wild sources. In addition to the observed poor effort of cultivating medicinal plants at home gardens, it was reported that most of the medicinal plants are under threat due to an ever-increasing anthropogenic influence on natural habitat of medicinal plants of the area. Deforestation (reported by 89% of informants); agricultural expansion (80%); charcoal and firewood (33%) and overgrazing (29%) were claimed to be the major factors affecting medicinal plant wealth of the area.

### Discussion and conclusions

Results showed that Ankober District is rich in medicinal plant diversity as shown by the presence of 135 species exhibiting wide taxonomic diversity (125 angiosperm, two gymnosperm and one fern genera in 68 angiosperm, two gymnosperm and one fern families). The diversity has also been made obvious in the elaborate system of traditional naming of plants (based on morphology of a plant part or its remedial uses) and the indigenous knowledge engraved in each medicinal plant species name and knowledge about the uses of each medicinal plant species. Results have also proved the role played by traditional medicinal plants and the local community holding considerable traditional health knowledge in assisting the primary healthcare needs of the District. The number of medicinal plants harvested in the District is found to be far higher than that of other areas in the country investigated for their ethnomedicinal wealth [24,25,34,53-55]. Although cultural, economic, ease of accessibility and efficacy related factors might have played major roles for the people of Ankober to rely on traditional medicine, the cultural factor might have been the most important one resulting in a sentimental adherence to the ancestral medical traditions/practices by upholding it as a highly valued heritage of the society or of the great fathers and mothers.

Dominance of medicinal plant species from families Asteraceae, Fabaceae, Solanaceae, Lamiaceae, Cucurbitaceae, Ranunculaceae and Rosaceae could be attributed to their wider distribution and abundance in the flora area [28,48,54]. This is also confirmed by consistent recording of ethnomedicinal uses of species from the aforementioned families in different Ethiopian ethnobotanical inventories [25,26,30,33,53]. Moreover, the wide utilization of species from these families might relate to the presence of effective bioactive ingredients against ailments [56].

Most medicinal plants used in the area (38%) were found to be herbs. This could relate to the fact that they are easily accessible in the nearby areas than trees and shrubs often harvested from patches of forests distantly located from resident areas. The finding agrees with the general pattern of dominance of herbaceous species seen in most medicinal plant inventories in Ethiopia and other countries [25,33,57-59]. Wild habitats of Ankober were found to be major pools of traditional medicinal plants providing about 74% of all reported medicinal plants. However, the investigation showed that these habitats are subjected to anthropogenic influences and consequently shrinking in size due to an ever-increasing population pressure resulting in the loss of many medicinal species sheltering in the wild. Our observation is also in agreement with previous reports of overdependence on wild habitats to harvest medicinal plants [26,34,35] than an effort to cultivate and use them sustainably.

Overexploitation of entire root parts for majority of medicinal plant preparations (44%) shows the threat posed on long-term survival of corresponding medicinal plants. Mining of root parts of medicinal plants was also commonly reported by other ethnomedicinal inventories elsewhere [8,26,30,33,60,61]. Harvesting of roots kills the parent plant and could be a severe threat for survival of the often rare and slowly reproducing medicinal plants of the area. As leaves of medicinal plant species were also reported to be harvested for most remedy preparations next to roots, gathering leaves could be promoted as a more sustainable method since in most cases at least a number of leaves are left over on the parent plant which then allows them to carry on its life functions.

Results also showed prominent use (69.9%) of freshly harvested plant parts for traditional remedy preparation used against various ailments. The recurrent use of freshly harvested medicinal plant materials in the area is reported to be related to the notion of attaining high efficacy using active ingredients of fresh plant parts which they thought could be lost on drying. Other ethnomedicinal inventories [26,33] have also indicated wide use of fresh plant materials for remedy preparations due to reportedly better efficacy related factors than using dried plant materials.

The significant difference (P < 0.05) on average number of medicinal plants reported by different age groups compared in this investigation showed that indigenous knowledge on use of medicinal plants is still strong with elderly people (4.90 ± 0.13 ) than in the younger generation (2.59 ± 0.08). Moreover, the observed extremely significant difference (p = 0.0001) showed the gap between generations and the decline of indigenous knowledge on medicinal plants down generations. This could be attributed to the impact of modernization (including urbanization and advent of formal education) and the very poor system of sharing indigenous knowledge (through word of mouth, with maximum secrecy and only along family lines) on medicinal plants to the younger generation. The scenario is the same for other cultural groups in Ethiopia [24,26,30,34] and elsewhere [6,31,62]. The output calls for an effort to close the observed generation gap through continuous professional support and training of local communities with an objective of preserving their traditional health knowledge and practices through systematic documentation. Silva *et al.*[6] explained that greater knowledge of older people on medicinal plants is the result of high degree of opportunity for more cultural contact and experience with plants and associated therapeutic uses than that of younger people. Absence of continuous cultural interaction with plants was also reported as one factor for loss of traditional knowledge down generation [2].

The other significant difference (p = 0.0001) observed between key and local; and literate and illiterate informants could relate to the impact of age-old experience and maximum degree of secrecy in using medicinal plants in the former, and modernization in the latter case. Similar results were reported by [24,30,63]. According to [64], community members who have greater contact with medicinal plants are more knowledgeable about therapeutic uses of the plants than those with intermittent contact.

Male informants of the District were found to report more medicinal plants on average (4.23 ± 0.13) than women (3.85 ± 0.19) even though the difference was not statistically significant (p = 0.1075). Thus, the result indicated that both men and women are knowledgeable on use of traditional plant remedies despite the relative dominance of medicinal plant tradition by men which could relate to the traditional flow of information along the male line in the country [30] and elsewhere [62,65]. Occurrence of relatively equivalent medicinal plants knowledge among men and women traditional medicine practitioners was reported by [4] for three communities in northeastern Brazil and by [66] for a community in southwest Niger. In contrast, [67] have reported the presence of more specialized knowledge on medicinal plants among women informants than men since they are often looked to diagnose and treat certain types of diseases. Generally, gender based differences in medicinal plant knowledge can be derived from experience and degree of cultural contact with curative plants [64].

The number and different types of diseases (69 disease types) for which traditional healers were most visited by patients indicated a preference of local people in the study area to visit traditional healers and the nature pharmacy. Economic, cultural, efficacy and availability factors were reported as the key factors which lead the community to knock at the door of traditional healthcare practitioners than the few distantly located healthcare centres with unaffordable prices. Similar findings were reported by [26,27,53].

Visual inspection of patients is the more obvious diagnostic method practiced by all local healers in the area. Although changes in body temperature, skin and eye colour, appetite and physical appearance help traditional healers to detect which patients face disorders it was only through visual experience that identification of diseases and prescriptions seem to be made. Other researchers [11,27,29,42,46,68] have also reported similar diagnostic methods in different cultural groups. Misidentification of diseases commonly leads to mis-prescription which may result in adverse effects to patients. Even though dosages of remedies for various ailments were reported to be determined based on age, occurrence of pregnancy, physical fitness/appearance and gender of the patient, there were no standardised measurements or guidelines set by traditional healers. Overdose of remedies was also reported to bring adverse effects like vomiting, diarrhoea, burning sensations and sometimes fainting of the patient. Lack of precision and standardization has been mentioned as a global drawback of the traditional healthcare system [22]. Traditional healers in our study area reported the use of different antidotes including BESSO, milk, coffee, honey, yoghurt, and butter for reversing adverse effects and stabilising any disorder. The same pattern of using antidotes was also reported for other cultural groups elsewhere [25,26,33].

The dominant use of medicinal plant decoctions for various ailments might be related to their proven effectiveness over many years of trial and indigenous knowledge accumulated on efficacy of such preparations. Decoction was also reported as one of the major ways of remedy preparation in ethnomedicinal inventory of other socio-cultural groups in the country [27,33].

The result from market survey of medicinal plants indicated that most medicinal plants (81%) have no marketability report and were not available on major market places of the District during the time of research. This would show that the majority of medicinal plants are collected from the wild for remedy preparations only when the need arises. Although 19% of the medicinal plants were available on the market *Echinops kebericho*, *Embelia schimperi*, *Hagenia abyssinica*, *Withania somnifera* and *Silene macrosolen* were the only ones to be sold or purchased for their traditional medicinal uses. The market value of these species (with a price range from 0.21 USD per bunch of root or jug of inflorescences to 0.3 USD for a cup of fruits) showed the income generation potential of a number of medicinal plants and gives an indication of potential demand of those marketable plants by the community. However, such marketability could also indicate that the plants are under pressure since they are purposefully hunted for economic reasons. Other reportedly marketable medicinal plants of Ankober were mainly gathered and sold for their uses related to edibility, lumbering, firewood and construction purposes. Although the investigation indicated current market potential of medicinal plants in Ankober, a relatively wider domestic trade of Ethiopian medicinal plants was reported for other cultural groups in the country [61,68-70]. Thus, our finding can also be used as a base line for a future in-depth study of the money-making potential of medicinal plants of the area through successive market survey over number of years and value chain analysis study of potential plants.

The highest recorded ICF values (0.7 and 0.65) indicated best agreement among informants’ on the use of medicinal plant species reported to be used for treating gastro-intestinal, and parasitic and dermatological diseases, respectively. The observed highest informants’ agreement coupled with high plant use citations for these disease categories could also indicate the relatively high incidence of the latter diseases in the area. According to [52], high ICF values are important to identify plants of particular interest in the search for bioactive compounds. Accordingly, about 21 medicinal plants of Ankober (with high ICF values) for treating gastro-intestinal and parasitic diseases are under investigation for their pharmacological properties by our research theme.

The reported highest fidelity level values for *Zehneria scabra* (95%) and *Ocimum lamiifolium* (93.33%) against febrile diseases; and *Hagenia abyssinica* (93.75%) against gastro-intestinal and parasitic diseases could be considered as a clue for the high healing potential of these plants against the corresponding diseases. Plants with highest fidelity level values could also be targeted for further phytochemical investigation to prove the bioactive components that are responsible for their high healing potential [52,55]. Accordingly, further activity testing experiments are being carried out on extracts of these species by our research group.

The output of a direct matrix ranking exercise showed highest values/ranks for a number of multipurpose medicinal plants of the study area including *Podocarpus falcatus*, *Olea europaea* and *Ekebergia capensis*. The result indicates that these plants are exploited more for their non-medicinal uses than for reported medicinal values. Overharvesting of multipurpose medicinal plant species for agricultural tool, construction, lumbering and firewood purposes were found the responsible factors aggravating depletion of the species in the area. Thus, the result calls for an urgent complementary conservation action to save the fast eroding multipurpose medicinal plant species of the area. Yineger *et al.*[33] has also reported the same pattern of highest exploitation of multipurpose medicinal plants for uses other than their traditional medicinal importance in south eastern Ethiopia.

The preference ranking exercise helped to identify the most-preferred medicinal plant species to treat atopic eczema. Accordingly, *Olea europaea* subsp. *cuspidata*, *Allium sativum* and *Datura stramonium* scored highest values and were found the most-preferred ones to treat the disease. Ethnobotanical investigation done elsewhere in Ethiopia [34] also reported the use of *Olea europaea* subsp. *cuspidata* for treating eczema. Further investigation of these species for their bioactive components against atopic eczema may bring promising results.

Lack of interest in traditional medicines was observed among the youngest generation of Ankober due to factors related to ‘modernization’. Similar findings were reported for other cultural groups in Ethiopia [24,26,71]. It was also found that traditional healers show maximum secrecy in handling medicinal plant knowledge. Moreover, they try not to leak the knowledge out of the family circle. These facts coupled with the absence of any written document on medicinal plants of the area show the threat on the future use of ethnomedicinal potential of Ankober.

Generally, although Ankober District was found to be rich in medicinal plant diversity, the effort to conserve the plants and associated indigenous knowledge was observed to be very poor. The effort from some traditional practitioners to cultivate medicinal plants at home gardens calls for a sustained governmental support to promote overall *in situ* and *ex situ* conservation strategies for medicinal plants of the District. It is also recommended to establish a traditional healers’ association in the District and strengthen members by providing professional support and land to establish as much medicinal plant nurseries as possible so as to conserve the fast-eroding medicinal plant wealth of the area.

## Competing interests

The authors declare that they have no competing interests.

## Authors’ contributions

All authors have equal contribution for this work and all have read and approved the final manuscript.

## Supplementary Material

Additional file 1: Appendix 1 List of medicinal plants used for human ailments: scientific name; family; local name; growth form; ailment treated; plant parts used; condition of plant part uses; methods of preparation and application, route of administration , plant part mixed with and voucher number. Click here for file
