# Supplementary material for: Ethnomedicinal study of plants used for human ailments in Ankober District, North Shewa Zone, Amhara Region, Ethiopia
Source: J Ethnobiol Ethnomed. 2013 Aug 28;9:63. doi: 10.1186/1746-4269-9-63 (PMC3846447; doi:10.1186/1746-4269-9-63)
Supplement: Additional file 1: Appendix 1 — List of medicinal plants used for human ailments: scientific name; family; local name; growth form; ailment treated; plant parts used; condition of plant part uses; methods of preparation and application, route of administration , plant part mixed with and voucher number. [file 1746-4269-9-63-S1.doc]

**Appendix 1 List of medicinal plants used for human ailments: scientific name; family; local name; growth form; ailment treated; plant parts used; condition of plant part uses; methods of preparation and application, route of administration , plant part mixed with and voucher number.**

Key: Growth form Tree (T); Shrub (S); Herb (H); Climber (C). Part used (Leaf, L; Root, R; Stem wood, St; Fruit, Fr; Bark, B; Root bark, RB; Stem bark, SB; Flower, Fl; Bulb, Bu; Rhizome, Rh; Latex, Lat). Conditions of part used (CPU) (Dry, D; Fresh, F). Methods of preparation and application (MPAP), 1. Boil and drink the decoction when cool; 2. Grind and paint the powder or crushed part ; 3. Grind, paste the crushed part and tie ; 4. Extract the juice/oil/latex and pour or paint it; 5. Crush, homogenize with cold water and drink; 6. Hold with teeth; 7. Crush, heat/ burn or boil the part and inhale its smoke or steam; 8. Crush and sniff the freshly crushed part; 9. Eat the part; 10. Boil and do steam bath; 11. Drink the concoction; 12. Boil the part and paint the decoction. Route of Administration; (RA) (Oral, O; Dermal, De; Nasal, Na; Optical, Op; Auricular, Au; Anal, An.)

| No | Scientific name | Family | Local name | Growth form | Ailment treated | Part used | CPU | MPAP | RA. | PMW | Voucher no  ErmiasL X | |
| --- | --- | --- | --- | --- | --- | --- | --- | --- | --- | --- | --- | --- |
| 1 | *Acacia abyssinica* Hochst. ex Benth. | Fabaceae | Girar | T | Chancroid | R | F/D | 1 | O |  | 826 |  |
| 2 | *Achyranthes aspera* L. | Amaranthaceae | Telenj | H | Minor bleeding | L | F | 3 | De |  | 104 |  |
|  |  |  |  |  | Omphalitis | L | F | 3 | De |  |  |  |
|  |  |  |  |  | Epistaxis | L | F | 4 | Na |  | 827 |  |
| 3 | *Acmella caulirhiza* Del. | Asteraceae |  | H | Gonorrhoea | Fl&L | F/D | 1 | O |  |  |  |
|  |  |  |  |  | Toothache | Fl | F | 6 | O |  |  |  |
|  |  |  |  |  | Epiglottitis | Fl&L | F | 1 | O |  |  |  |
|  |  |  |  |  | Impotence | Fl&L | F | 1 | O |  |  |  |
|  |  |  |  |  | Tonsillitis | Fl | F | 1 | O |  |  |  |
| 4 | *Acokanthera schimperi* (A.DC.) Schweinf. | Apocynaceae | Merenz | S | Gonorrhoea | R | F | 1 | O |  | 100 |  |
| 5 | *Allium sativum* L.* | Alliaceae | Nech Shinkurt | H | Herpes zoster | Bu | F | 4 | De | 24 | 828 |  |
|  |  |  |  |  | Atopic eczema | Bu | F | 4 | De |  |  |  |
|  |  |  |  |  | Ringworm | Bu | F | 4 | De |  |  |  |
|  |  |  |  |  | Asthma | Bu | F/D | 11 | O | 91 |  |  |
|  |  |  |  |  | Common cold | Bu | F/D | 1 | O |  |  |  |
|  |  |  |  |  | Dandruff | Bu | F | 4 | De |  |  |  |
|  |  |  |  |  | Pneumonia | Bu | F/D | 1 | O |  |  |  |
|  |  |  |  |  | Coughing | Bu | F/D | 11 | O |  |  |  |
|  |  |  |  |  | Malaria | Bu | F/D | 9 | O |  |  |  |
| 6 | *Allophyllus abyssinicus* (Hochst.) Radlk. | Sapindaceae | Imbis | T | Hepatitis | R | F | 1 | O |  | 829 |  |
| 7 | *Anthemis tigreensis* J. Gay ex A. Rich. | Asteraceae |  | H | Rabies | R | F | 1 | O | 9 | 830 |  |
|  |  |  |  |  | Evil spirit | R | F/D | 7 | Na |  |  |  |
| 8 | *Arisaema schimperianum* Schott. | Araceae | Amoch | H | Scabies | R | F | 2 | De |  | 587 |  |
|  |  |  |  |  | Ringworm | R | F | 2 | De |  |  |  |
|  |  |  |  |  | Skin lesion | R | F | 2 | De |  |  |  |
| 9 | *Asparagus africanus* Lam. | Asparagaceae | Yeset Kest | S | Impotence | R | F/D | 1 | O |  | 189 |  |
|  |  |  |  |  | Gonorrhoea | R | F/D | 1 | O | 4 |  |  |
|  |  |  |  |  | Scabies | R | F | 2 | De |  |  |  |
|  |  |  |  |  | Atopic eczema | R | F | 2 | De |  |  |  |
|  |  |  |  |  | Epilepsy | R,L,SB | F/D | 7 | Na |  |  |  |
|  |  |  |  |  | Bone fracture | St | F | 3 | De |  |  |  |
|  |  |  |  |  | Chancroid | R | F | 1 | O | 4 |  |  |
|  |  |  |  |  | Skin lesion | R | F | 2 | De |  |  |  |
| 10 | *Asplenium aethiopicum* (Kunth) mett. | Aspleniaceae | | H | Headache | R | F | 7 | Na |  | 593 |  |
|  |  |  |  |  | Epilepsy | R, L | F | 7 | Na |  |  |  |
| 11 | *Berberis holstii* Engl. | Berberidaceae | Yeset Af | H | Hemorrhoids | R | F | 4 | De | 82 | 831 |  |
|  |  |  |  |  | Jaundice | R | F | 1 | O |  |  |  |
| 12 | *Bersama abyssinica* Fresen. | Melianthaceae | Azamir | T | Diarrhoea | L | F | 1 | O |  | 173 |  |
|  |  |  |  |  | Constipation | L | F | 1 | O |  |  |  |
|  |  |  |  |  | Ascariasis | Se | F | 1 | O |  |  |  |
| 13 | *Buddleja polystachya* Fresen. | Loganiaceae | Anfar | S | Evil eye | R | F | 7 | Na |  | 542 |  |
| 14 | *Cassipourea malosana* (Baker) Alston | Rhizophoraceae | Werer | T | Menstrual disorder | SB | F | 1 | O |  | 832 |  |
| 15 | *Calpurnia aurea* (Ait.) Benth. | Fabaceae | Digita | S | Diarrhoea | R | F | 1 | O |  | 76 |  |
|  |  |  |  |  | Constipation | R | F | 1 | O |  |  |  |
|  |  |  |  |  | Scabies | L | F | 2 | De |  |  |  |
|  |  |  |  |  | Toothache | R | F | 6 | O |  |  |  |
|  |  |  |  |  | Ascariasis | L | F | 1 | O |  |  |  |
|  |  |  |  |  | Sore | R | F | 4 | De |  |  |  |
|  |  |  |  |  | Epiglottitis | R | F | 1 | O |  |  |  |
|  |  |  |  |  | Giardiasis | R | F | 1 | O |  |  |  |
| 16 | *Capsicum frutescens* L.* | Solanaceae | Mitmita | S | Retained placenta | R | F/D | 1 | O |  | 204 |  |
| 17 | *Carica papaya* L.* | Caricaceae | Papaya | T | Taeniasis | Se | F | 9 | O |  | 40 |  |
|  |  |  |  |  | Dryness abdominal | Se | F | 9 | O |  |  |  |
|  |  |  |  |  | Gastritis | Se | F | 9 | O |  |  |  |
|  |  |  |  |  | Constipation | se | F | 9 | O |  |  |  |
| 18 | *Carissa spinarum* L. | Apocynaceae | Agam | S | Diarrhoea | R | F | 5 | O |  | 16 |  |
|  |  |  |  |  | Constipation | R | F | 1 | O |  |  |  |
|  |  |  |  |  | Taeniasis | R | F/D | 5 | O |  |  |  |
|  |  |  |  |  | Evil spirit | R | F/D | 7 | Na |  |  |  |
|  |  |  |  |  | Headache | L | F | 7 | Na |  |  |  |
|  |  |  |  |  | Snake bite | R | F | 1 | O |  |  |  |
|  |  |  |  |  | Rheumatism | R | F | 1 | O |  |  |  |
| 19 | *Catha edulis* ( Vahl) Forssk. ex Endl.* | Celasteraceae | Chat | S | Chancroid | R | F/D | 1 | O |  | 26 |  |
|  |  |  |  |  | Impotence | R | F/D | 1 | O |  |  |  |
| 20 | *Celtis africana* Burm.f. | Ulmaceae | Kewt | T | Evil eye | RB | F | 7 | Na |  | 833 |  |
|  |  |  |  |  | Rheumatism | L | F | 4 | De | 44 |  |  |
| 21 | *Chamaecrista mimosoides* (L.) Greene | Fabaceae | Yeayt Gomen | H | Cystitis | L | F | 1 | O |  | 834 |  |
| 22 | *Chenopodium ambroisoides* L. | Chenopodiaceae | Gundan Abir | S | Evil eye | L | F | 8 | Na |  | 4 |  |
| 23 | *Citrus aurantifolia* (L.) Burm.f.* | Rutaceae | Lomi | S | Epistaxis | Fr & L | F | 4 | Na |  | 155 |  |
|  |  |  |  |  | Minor bleeding | Fr & L | F | 4 | De |  |  |  |
| 24 | *Clematis hirsuta* Perr. & Guill. | Ranunculaceae | Azo Hareg | C | Diarrhoea | L | F | 5 | O |  | 18 |  |
|  |  |  |  |  | Taeniasis | R | F/D | 5 | O |  |  |  |
|  |  |  |  |  | Constipation | R | F/D | 5 | O |  |  |  |
|  |  |  |  |  | Atopic eczema | L | F | 4 | De |  |  |  |
|  |  |  |  |  | Mumps | L | F | 4 | Au |  |  |  |
|  |  |  |  |  | Ringworm | L | F | 4 | De |  |  |  |
|  |  |  |  |  | Herpes zoster | L | F | 4 | De | 5 |  |  |
|  |  |  |  |  | Otorrhea | L | F | 4 | Au |  |  |  |
|  |  |  |  |  | Conjunctivitis | L | F | 4 | Op |  |  |  |
|  |  |  |  |  | Gastritis | R | F/D | 5 | O |  |  |  |
|  |  |  |  |  | Leishmaniasis | L | F | 4 | De |  |  |  |
| 25 | *Clutia abyssinica* Jaub. and Spach. | Euphorbiaceae | Fiyele Fej | S | Bloody diarrhoea | R | F/D | 1 | O |  | 10 |  |
|  |  |  |  |  | Taeniasis | R | F/D | 1 | O |  |  |  |
|  |  |  |  |  | Constipation | R | F/D | 1 | O |  |  |  |
|  |  |  |  |  | Fever | L | F | 1 | O |  |  |  |
|  |  |  |  |  | Gastritis | R | F/D | 1 | O |  |  |  |
|  |  |  |  |  | Malaise | L | F | 1 | O |  |  |  |
| 26 | *Coffea arabica* L.* | Rubiaceae | Bunna | S | Chancroid | R | F | 1 | O |  | 12 |  |
| 27 | *Crassocephalum macropapum* (Sch. Bip. ex A. Rich.) S. Moore** | Asteraceae |  | H | Evil spirit | R | F/D | 7 | Na |  | 835 |  |
| 28 | *Crotalaria incana* L. | Fabaceae |  | S | Wound | R | F | 3 | De |  | 836 |  |
| 29 | *Croton macrostachyus* Del. | Euphorbiaceae | Bisana | T | Tinea versicolor | L | F | 4 | De |  | 17 |  |
|  |  |  |  |  | Atopic eczema | L | F | 4 | De |  |  |  |
|  |  |  |  |  | Sore | L | F | 2 | De |  |  |  |
|  |  |  |  |  | Allergic rushes on skin | L | F | 4 | De |  |  |  |
| 30 | *Cucumis ficifolius* A. Rich. | Cucurbitaceae | Yemdr Imbuay | H | Evil spirit | L | F/D | 7 | Na |  | 68 |  |
|  |  |  |  |  | Rabies | Fr | F | 1 | O |  |  |  |
| 31 | *Cucurbita pepo*L.* | Cucurbitaceae | Duba | H | Hepatitis | Se | F/D | 1 | O |  | 96 |  |
| 32 | *Cyathula cylindrica* Moq. | Amaranthaceae | Yedem Abnet | H | Epistaxis | L | F | 4 | Na |  | 837 |  |
|  |  |  |  |  | Minor bleeding | L | F | 3 | De |  |  |  |
| 33 | *Cymbopogon citratus* (DC ex Nees) Stapf* | Fabaceae | Tej Sar | H | Chicken pox | L | F/D | 10 | De |  | 838 |  |
| 34 | *Cynoglossum coeruleum* Hochst. | Boraginaceae | Chegogot | H | Fever | R, L | F | 1 | O |  | 66 |  |
|  |  |  |  |  | Malaise | R, L | F | 1 | O |  |  |  |
| 35 | *Cyperus bulbosus* Vahl | Cyperaceae | Ingicha | H | Jaundice | R | F | 5 | O |  | 839 |  |
| 36 | *Datura stramonium* L. | Solanaceae | Astenagir | S | Dandruff | L | F | 2 | De |  | 241 |  |
|  |  |  |  |  | Atopic eczema | L | F | 2 | De |  |  |  |
|  |  |  |  |  | Skin lesion | L | F | 2 | De |  |  |  |
| 37 | *Desmodium repandum* (V ahl) DC. | Fabaceae |  | H | Epilepsy | R | F/D | 7 | Na |  | 840 |  |
| 38 | *Desmodium velutinum* (Willd.) DC. | Fabaceae |  | H | Snake bite | R | F | 1 | O |  | 11 |  |
| 39 | *Discopodium penninervium* Hochst. | Solanaceae | Ameraro | S | Snake bite | R | F | 1 | O |  | 606 |  |
| 40 | *Dodonaea angustifolia* L. f. | Sapindaceae | Kitkita | S | Diarrhoea | R | F | 1 | O |  | 20 |  |
|  |  |  |  |  | Taeniasis | R | F/D | 1 | O |  |  |  |
|  |  |  |  |  | Constipation | R | F/D | 1 | O |  |  |  |
|  |  |  |  |  | Ringworm | R | F | 2 | De |  |  |  |
|  |  |  |  |  | Skin lesion | R | F | 2 | De |  |  |  |
|  |  |  |  |  | Sore | L | F | 3 | De |  |  |  |
| 41 | *Dombeya torrida* (J. F. Gmel.) P. Bamps | Sterculariaceae | Wulkifa | T | Snake bite | R | F | 1 | O |  | 841 |  |
| 42 | *Dovyalis abyssinica* (A. Rich.) Warb. | Flacourtiaceae | Koshim | S | Menstrual disorder | R | F/D | 1 | O |  | 536 |  |
| 43 | *Draceana studenri* Engl. | Dracenaceae | Itse Patos | T | Hepatitis | R | F/D | 1 | O |  | 426 |  |
| 44 | *Dregea schimperi* (Dec.) Bull. | Asclepiadaceae | | C | Rheumatism | R | F | 4 | De | 20 | 798 |  |
|  |  |  |  |  | Gout | R | F | 4 | De |  |  |  |
| 45 | *Echinops kebericho* Mesfin** | Asteraceae | Kebericho | S | Evil eye | R | F/D | 7 | Na |  | 842 |  |
|  |  |  |  |  | Evil spirit | R | F/D | 7 | Na |  |  |  |
|  |  |  |  |  | Malaise | R | F | 1 | O |  |  |  |
|  |  |  |  |  | Fever | R | F | 1 | O |  |  |  |
| 46 | *Ehertia cymosa* Thonn. | Boraginaceae | Game | T | Retained placenta | R | F | 1 | O |  | 843 |  |
| 47 | *Ekebergia capensis* Sparrm. | Meliaceae | Ilol | T | Menstrual disorder | SB | F/D | 1 | O |  | 844 |  |
| 48 | *Embelia schimperi* Vatke | Myrsinaceae | Inkoko | T | Diarrhoea | Fr | F/D | 5 | O |  | 505 |  |
|  |  |  |  |  | Taeniasis | Fr | F/D | 5 | O |  |  |  |
|  |  |  |  |  | Constipation | Fr | F/D | 5 | O |  |  |  |
|  |  |  |  |  | Ascariasis | Fr | F/D | 5 | O |  |  |  |
| 49 | *Epilobium hirsutum* L. | Onagraceae | Limich | H | Chicken pox | R | F | 4 | De |  | 845 |  |
| 50 | *Erica arborea* L. | Ericaceae | Asta | S | Lymphadenopathy | R | F | 1 | O |  | 846 |  |
| 51 | *Erythrina brucei* Schwinef.** | Fabaceae | Korch | T | Stabbing pain | R | F | 1 | O |  | 247 |  |
| 52 | *Eucalyptus globulus* Labill. | Myrtaceae | Bahir Zaf | T | Common cold | L | F | 7 | Na |  | 847 |  |
|  |  |  |  |  | Coughing | L | F | 7 | Na |  |  |  |
|  |  |  |  |  | Pneumonia | L | F | 7 | Na |  |  |  |
|  |  |  |  |  | Gout | L | F | 10 | De |  |  |  |
|  |  |  |  |  | Headache | L | F | 7 | Na |  |  |  |
| 53 | *Euclea divinorum* Hiern | Ebenaceae | Dedeho | T | Gonorrhoea | R | F/D | 1 | O |  | 14 |  |
|  |  |  |  |  | Pyelonephritis | SB | F | 1 | O |  |  |  |
|  |  |  |  |  | Impotence | R | F/D | 1 | O |  |  |  |
|  |  |  |  |  | Malaise | R | F | 10 | De |  |  |  |
|  |  |  |  |  | Fever | R | F | 10 | De |  |  |  |
|  |  |  |  |  | Chancroid | R | F/D | 1 | O |  |  |  |
| 54 | *Ficus sur* Forssk. | Moraceae | Shola | T | Impotence | R | F | 4 | De |  | 822 |  |
| 55 | *Foeniculum vulgare* Miller* | Apiaceae | Insilal | H | Epistaxis | L | F | 4 | Na |  | 848 |  |
|  |  |  |  |  | Lymphadenopathy | L | F | 4 | De |  |  |  |
| 56 | *Galineria saxifraga*(Hochst.) Bridson | Rubiaceae | Tota Kula | T | Pyelonephritis | R | F | 1 | O |  | 849 |  |
|  |  |  |  |  | Gonorrhoea | R | F/D | 1 | O |  |  |  |
| 57 | *Galium aparinoides* Forssk. | Rubiaceae | Ashkit | H | Lymphadenopathy | R | F | 1 | O |  | 884 |  |
| 58 | *Geranium arabicum* Forssk. | Geraniaceae | | H | Evil eye | R | F | 1 | O |  | 883 |  |
| 59 | *Gomphocarpus fruticosus* (L.) Ait. F. | Asclepiadaceae | Ash Hareg | C | Gout | R | F | 1 | O | 66 | 133 |  |
| 60 | *Guizotia abyssinica* Cass* | Asteraceae | Noug | H | Common Cold | Se | F/D | 1 | O |  | 882 |  |
|  |  |  |  |  | Asthma | Se | F/D | 1 | O |  |  |  |
|  |  |  |  |  | Coughing | Se | F/D | 1 | O |  |  |  |
| 61 | *Guizotia scabra* (Vis.) Chiov. | Asteraceae | Mech | H | Conjunctivitis | Fl | F | 4 | Op |  | 878 |  |
|  |  |  |  |  | Mumps | Fl | F | 4 | Au |  |  |  |
| 62 | *Hagenia abyssinica* (Bruce) J.F. Gmel. | Rosaceae | Koso | T | Taeniasis | Fl | F/D | 5 | O |  | 598 |  |
|  |  |  |  |  | Ascariasis | Fl | F/D | 5 | O |  |  |  |
|  |  |  |  |  | Amoebiasis | Fl | F/D | 5 | O |  |  |  |
|  |  |  |  |  | Constipation | Fl | F/D | 5 | O |  |  |  |
| 63 | *Halleria lucida* L. | Scrophulariaceae | Mesenqero | T | Evil eye | R | F | 7 | Na |  | 880 |  |
| 64 | *Haplocarpha schimperi* (Sch. Bip.) Beauv. | Asteraceae | Getim | H | Hemorrhoids | R | F | 4 | An |  | 879 |  |
|  |  |  |  |  | Lymphadenopathy | R | F | 4 | De |  |  |  |
| 65 | *Heteromorpha arborescens* (Spreng.) Cham. & Schlecht. | Apiaceae |  | H | Evil spirit | R | F/D | 7 | Na |  | 30 |  |
| 66 | *Hypericum revolutum* Vahl | Hypericaceae | Amja | T | Otorrhea | Fl | F | 4 | Au |  | 872 |  |
|  |  |  |  |  | Mumps | Fl | F | 4 | Au |  |  |  |
| 67 | *Hypoestes aristata*(Vahl) Soland. | Acanthaceae | Telenj | H | Gout | L | F | 12 | De |  | 599 |  |
|  |  |  |  |  | Headache | R | F/D | 7 | Na |  |  |  |
| 68 | *Hypoestes forskaolii* (Vahl) R. Br. | Acanthaceae | Tay Beder | H | Anaemia | L | F | 1 | O |  | 506 |  |
| 69 | *Impatiens tinctoria* A. Rich. | Balsaminaceae | Gishrit | H | Gout | R | F | 12 | De |  | 620 |  |
|  |  |  |  |  | Rheumatism | R | F | 12 | De |  |  |  |
|  |  |  |  |  | Dandruff | R | F | 12 | De |  |  |  |
|  |  |  |  |  | Tinea versicolor | R | F | 12 | De |  |  |  |
|  |  |  |  |  | Skin infection | R | F | 12 | De |  |  |  |
| 70 | *Inula confertiflora* A. Rich.** | Asteraceae | Woynagift | S | Leprosy | Fl | F | 4 | De |  | 877 |  |
|  |  |  |  |  | Asthma | L | F | 1 | O | 5 |  |  |
|  |  |  |  |  | Common cold | L | F | 1 | O |  |  |  |
|  |  |  |  |  | Coughing | L | F | 1 | O |  |  |  |
| 71 | *Ipomea obscura* (L.) ker-Gawl. | Convolvulaceae | | H | Hemorrhoids | L | F | 2 | An |  | 876 |  |
| 72 | *Jasminum abyssinicum* Hochst. | Oleaceae | Abita | c | Bloody diarrhoea | L | F | 1 | O |  | 577 |  |
|  |  |  |  |  | Taeniasis | L | F | 5 | O |  |  |  |
|  |  |  |  |  | Gastritis | L | F | 5 | O |  |  |  |
|  |  |  |  |  | Constipation | Fl | F | 5 | O |  |  |  |
|  |  |  |  |  | Wound | R | F | 12 | De |  |  |  |
| 73 | *Jasminum grnadiflorum* L. | Oleaceae |  | C | Hemorrhoids | Fl | F | 2 | An | 76, 99 | 875 |  |
|  |  |  |  |  | Jaundice | Fl | F | 1 | O |  |  |  |
|  |  |  |  |  | Mumps | L | F | 4 | Au |  |  |  |
|  |  |  |  |  | Conjunctivitis | L | F | 4 | Op |  |  |  |
| 74 | *Juniperus procera* L. | Cuprussaceae | Yeabesha Tsid | T | Evil spirit | R | F/D | 7 | Na |  | 874 |  |
| 75 | *Justicia schimperiana* (Hochst. ex Nees) T. Anders. | Acanthaceae | Sensel | S | Epiglottitis | R | F | 1 | O |  | 506 |  |
|  |  |  |  |  | Tonsillitis | R | F | 1 | O |  |  |  |
| 76 | *Kalanchoe petitiana* A. Rich. | Crassulaceae | Indahula | H | Hemorrhoids | L | F | 2 | An | 73,99 | 94 |  |
|  |  |  |  |  | Bone fracture | L | F | 4 | De |  |  |  |
| 77 | *Kniphofia foliosa* Hochst.** | Asphodelaceae | Abelbila | S | Evil spirit | R | D | 7 | Na |  | 869 |  |
| 78 | *Laggera tomentosa* Sch.-Bip.** | Asteraceae | Keskeso | S | Hemorrhoids | R | F | 2 | An |  | 881 |  |
| 79 | *Leonotis ocymifolia* (Burm.f.) Iwarsson | Lamiaceae | Ras Kimir | S | Nausea | L | F | 5 | O |  | 871 |  |
| 80 | *Lepidium sativum* L.* | Brassicaceae | Feto | H | Malaise | Se | F/D | 1 | O |  | 870 |  |
|  |  |  |  |  | Tonsillitis | Se | F/D | 5 | O |  |  |  |
|  |  |  |  |  | Gingivitis | Se | F/D | 6 | O |  |  |  |
|  |  |  |  |  | Fever | Se | F/D | 1 | O |  |  |  |
|  |  |  |  |  | Malaise | Se | F/D | 2 | De |  |  |  |
|  |  |  |  |  | Epiglottitis | Se | F/D | 1 | O |  |  |  |
|  |  |  |  |  | Wound | Se | F/D | 3 | De |  |  |  |
| 81 | *Linum usitatissimum* L.* | Linaceae | Telba | H | Retained placenta | Se | F/D | 5 | O |  | 873 |  |
| 82 | *Lobelia gibberroa* Hemsl. | Lobeliaceae | Gibira | S | Topical ulcer | Lat | F | 4 | De | 11 | 554 |  |
|  |  |  |  |  | Tinea versicolor | Lat | F | 4 | De |  |  |  |
|  |  |  |  |  | Skin lesion | Lat | F | 4 | De |  |  |  |
| 83 | *Maesa lanceolata* Forssk. | Myrsinaceae | Kelewa | S | Diarrhoea | L | F | 1 | O |  | 42 |  |
|  |  |  |  |  | Constipation | R | F/D | 1 | O |  |  |  |
|  |  |  |  |  | Taeniasis | Fr | F | 5 | O |  |  |  |
| 84 | *Maytenus arbutifolia* (A. Rich.) Wilczek | Celastraceae | Atat | T | Lymphadenopathy | R | F | 1 | O |  | 47 |  |
| 85 | *Maytenus undata* (Thunb.) Blakelock | Celastraceae | Damot woyra | T | Headache | R | F | 7 | Na |  | 868 |  |
| 86 | *Microglossa pyrifolia* (Lam.) Kuntze | Asteraceae |  | H | Menstrual disorder | L | F | 1 | O |  | 144 |  |
| 87 | *Momordica foetida* Schumach. | Cucurbitaceae | Yamora Misa | S | Constipation | R | F | 1 | O |  | 867 |  |
|  |  |  |  |  | Malaise | R, L | F | 10 | De |  |  |  |
|  |  |  |  |  | Gastritis | R | F | 1 | O |  |  |  |
|  |  |  |  |  | Taeniasis | Fr | F/D | 1 | O |  |  |  |
|  |  |  |  |  | Fever | R, L | F | 10 | De |  |  |  |
|  |  |  |  |  | Ascariasis | Fr | F/D | 5 | O |  |  |  |
| 88 | *Myrica salicifolia* A. Rich. | Myricaceae |  | S | Vomiting and Nausea | R | F | 1 | O |  | 866 |  |
| 89 | *Myrsine africana* L. | Myrsinaceae | Kechemo | T | Diabetis mellitus | Fr | F | 5 | O |  | 49 |  |
| 90 | *Nicotiana tabacum* L. * | Solanaceae | Timbaho | S | Epilepsy | R, L | F | 8 | Na |  | 246 |  |
| 91 | *Nigella sativa* L.* | Ranunculaceae | Tikur Azmud | H | Asthma | Se | F/D | 11 | O | 5 | 865 |  |
| 92 | *Ocimum lamiifolium* Hochst.* | Lamiaceae | Dama kesse | H | Malaise | L | F | 5 | O |  | 864 |  |
|  |  |  |  |  | Otorrhea | L | F | 4 | Au |  |  |  |
|  |  |  |  |  | Conjunctivitis | L | F | 4 | Op |  |  |  |
|  |  |  |  |  | Fever | L | F | 12 | De |  |  |  |
| 93 | *Ocimum urticifolium* Roth | Lamiaceae | Dama kesse | H | Headache | L | F | 5 | O |  | 69 |  |
| 94 | *Olea europaea* L. subsp. *cuspidata* (Wall. ex G.Don) | Oleaceae | Woyra | T | Leprosy | Fr | F | 4 | De |  | 19 |  |
|  |  |  |  |  | Atopic eczema | Fr | F | 4 | De |  |  |  |
|  |  |  |  |  | Mumps | Fr | F | 4 | Au |  |  |  |
|  |  |  |  |  | Tonsillitis | Fr | F | 4 | O |  |  |  |
|  |  |  |  |  | Ringworm | St & L | F | 4 | De |  |  |  |
|  |  |  |  |  | Tinea versicolor | St & L | F | 4 | De |  |  |  |
|  |  |  |  |  | Otorrhea | Fr | F | 4 | Au |  |  |  |
|  |  |  |  |  | Toothache | St | F | 6 | O |  |  |  |
| 95 | *Olinia rochetiana* A. Juss. | Oliniaceae | Tife | T | Diarrhoea | R | D | 1 | O |  | 102 |  |
|  |  |  |  |  | Constipation | R | D | 1 | O |  |  |  |
|  |  |  |  |  | Epilepsy | R | F/D | 7 | Na |  |  |  |
| 96 | *Opuntia ficus-indica* (L.) Miller | Cactaceae | Beles | S | Cystitis | St | F | 1 | O |  | 863 |  |
| 97 | *Osyris quadripartita* Decn. | Santalaceae | Keret | S | Topical ulcer | R | F | 2 | De |  | 211 |  |
|  |  |  |  |  | Tinea versicolor | R | F | 2 | De |  |  |  |
| 98 | *Pennisetum sphacelatum* (Nees) Th. Dur. & Schinz | Poaceae | Seged achawach | H | Gout | L | F | 12 | De |  | 862 |  |
| 99 | *Periploca linearifolia* Quaart.-Dill. & A. Rich. | Asclepiadaceae | Imbouayder | C | Hemorrhoids | R | F | 4 | An | 73, 76 | 83 |  |
|  |  |  |  |  | Hemorrage | R | F | 3 | De |  |  |  |
| 100 | *Persicaria senegalensis* (Meisn.) Miyabe | Polygonaceae | Aluma | H | Jaundice | L | F | 1 | O |  | 861 |  |
| 101 | *Phytolacca dodecandra* L' Herit. | Phytolaccaceae | Indod | S | Gonorrhoea | R | D | 1 | O |  | 243 |  |
|  |  |  |  |  | Syphilis | R | D | 1 | O |  |  |  |
|  |  |  |  |  | Pyelonephritis | R | D | 1 | O |  |  |  |
| 102 | *Plantago lanceolata* L. | Plantaginaceae | Wusha milas | H | Hemorrage | L | F | 3 | De |  | 860 |  |
| 103 | *Plectranthus lactiflorus* (L.) Agnew | Lamiaceae | Ayderkush | H | Rabies | L | F/D | 11 | O | 101 | 859 |  |
|  |  |  |  |  | Menstrual disorder | R, L | F | 12 | De |  |  |  |
| 104 | *Podocarpus falcatus* (Thunb.) Mirb. | Podocarpaceae | Zigba | T | Evil spirit | R | D | 7 | Na |  | 197 |  |
|  |  |  |  |  | Bone fracture | R | F | 3 | De | 131 |  |  |
| 105 | *Premna schimperii* Engl. | Lamiaceae | Chocho | S | Evil spirit | R | F/D | 7 | Na |  | 202 |  |
| 106 | *Prunus africana* (Hook. f.) Kalkm. | Rosaceae | Tikur Inchet | T | Syphilis | RB | F | 1 | O |  | 439 |  |
|  |  |  |  |  | Gonorrhoea | RB | F | 1 | O |  |  |  |
| 107 | *Pterolobium stellatum* (Forssk.) Brenan | Fabaceae | Kontir | S | Evil eye | R | F | 7 | Na |  | 46 |  |
| 108 | *Ranunculus multifidus* Forssk | Ranunculaceae | Etse siol | H | Hemorrhoids | R | F | 2 | An |  | 858 |  |
|  |  |  |  |  | Snake bite | R | F/D | 5 | O |  |  |  |
|  |  |  |  |  | Lymphadenopathy | R | F | 4 | De |  |  |  |
|  |  |  |  |  | Jaundice | R | F/D | 5 | O |  |  |  |
|  |  |  |  |  | Wound | L | F | 3 | De |  |  |  |
| 109 | *Rhamnus prinoides* L'Herit.* | Rhamnaceae | Gesho | S | Epiglottitis | L | F | 4 | O |  | 607 |  |
|  |  |  |  |  | Tonsillitis | L | F | 4 | O |  |  |  |
| 110 | *Rhus vulgaris* Meikle | Anacardiaceae | Kimo | T | Retained placenta | R | F | 1 | O |  | 857 |  |
| 111 | *Ricinus communis* L.* | Euphorbiaceae | Gulo | S | Hemorrhoids | Fr | F | 4 | An |  | 33 |  |
|  |  |  |  |  | Rheumatism | Fr | F | 12 | De |  |  |  |
| 112 | *Rosa abyssinica* Lindley | Rosaceae | Kega | S | Jaundice | R | F | 1 | O |  | 856 |  |
|  |  |  |  |  | Constipation | R | F | 1 | O |  |  |  |
| 113 | *Rubus steudneri* Schweinf. | Rosaceae | Injori | S | Diarrhoea | R | F | 5 | O |  | 242 |  |
|  |  |  |  |  | Gastritis | R | F | 5 | O |  |  |  |
|  |  |  |  |  | Constipation | R | F/D | 5 | O |  |  |  |
| 114 | *Rumex nepalensis* Spreng. | Polygonaceae | Lut | H | Diarrhoea | R | F/D | 1 | O |  | 24 |  |
|  |  |  |  |  | Constipation | R | F/D | 1 | O |  |  |  |
| 115 | *Ruta chalepensis* L.* | Rutaceae | Tena adam | S | Common Cold | Fr & L | F | 1 | O |  | 37 |  |
| 116 | *Scabiosa columbaria* L. | Dipsacaceae | | H | Stabbing pain | R, L | F | 1 | O |  | 855 |  |
| 117 | *Sida schimperiana* Hochst. ex A. Rich. | Malvaceae | Garda | S | Fever | R | F | 1 | O |  | 153 |  |
| 118 | *Silene macrosolen* steud ex. Rich | Caryophyllaceae | Wegert | H | Headache | R | F/D | 7 | Na |  | 854 |  |
|  |  |  |  |  | Evil eye | R | F/D | 7 | Na |  |  |  |
|  |  |  |  |  | Chancroid | R | F | 1 | O |  |  |  |
|  |  |  |  |  | Impotence | R | F | 1 | O |  |  |  |
|  |  |  |  |  | Gonorrhoea | R | F | 1 | O |  |  |  |
| 119 | *Smilax aspera* L. | Smilacaceae | Ashkla hareg | C | Nausea | Fr | F | 5 | O |  | 853 |  |
|  |  |  |  |  | Hepatitis | Fr | F | 5 | O |  |  |  |
| 120 | *Solanecio gigas* (Vatke) C. Jeffrey** | Asteraceae | Dengorita | T | Evil spirit | R | F | 7 | Na |  | 531 |  |
| 121 | *Solanum anguivi* Lam. | Solanaceae | Imbuay | S | Scabies | Fr | F | 4 | De |  | 565 |  |
|  |  |  |  |  | Dandruff | Fr | F | 4 | De |  |  |  |
|  |  |  |  |  | Atopic eczema | Fr | F | 4 | De |  |  |  |
| 122 | *Solanum marginatum* L.f. | Solanaceae | Imbuay | S | Rabies | R | D | 1 | O |  | 205 |  |
| 123 | *Stephania abyssinica* (Dill. & A. Rich.) Walp. | Menispermaceae | Yeait hareg | H | Evil eye | R | F | 7 | Na |  | 130 |  |
|  |  |  |  |  | Malaise | R | F | 1 | O |  |  |  |
| 124 | *Thalictrum rhynchocarpum* Dill. & Rich. | Ranunculaceae | Sire bizu | H | Diarrhoea | R | F | 5 | O |  | 535 |  |
|  |  |  |  |  | Taeniasis | R | F/D | 5 | O |  |  |  |
|  |  |  |  |  | Mumps | R | F | 4 | Au |  |  |  |
|  |  |  |  |  | Ascariasis | R | F/D | 5 | O |  |  |  |
|  |  |  |  |  | Constipation | R | F/D | 5 | O |  |  |  |
|  |  |  |  |  | Otorrhea | R | F | 4 | Au |  |  |  |
| 125 | *Thymus schimperi* Ronniger | Lamiaceae | Tosigne | H | Hypertension | L | F | 1 | O |  | 826 |  |
| 126 | *Toddalia asiatica* Lam. | Rutaceae | Gumare | T | Hepatitis | Fl | F | 1 | O |  | 97 |  |
| 127 | *Trifolium semipilosum* Fresen. | Fabaceae |  | H | Hepatitis | R | F/D | 5 | O |  | 825 |  |
| 128 | *Urera hypselodendron* (A. Rich.) Wedd. | Urticaceae | Lankuso | C | Chancroid | R, L | F | 1 | O |  | 538 |  |
|  |  |  |  |  | Cystitis | R | F/D | 5 | O |  |  |  |
| 129 | *Urtica dioica* L. | Urticaceae | Sama | H | Chicken pox | L | D | 9 | De |  | 824 |  |
| 130 | *Verbascum sinaiticum* Benth. in DC. | Scrophulariaceae | Yeahya Joro | H | Diarrhoea | L | F | 1 | O |  | 852 |  |
|  |  |  |  |  | Constipation | R | F/D | 5 | O |  |  |  |
| 131 | *Vernonia amygdalina* Del. | Asteraceae | Girawa | T | Diarrhoea | L | F | 1 | O |  | 22 |  |
|  |  |  |  |  | Taeniasis | L | F | 5 | O |  |  |  |
|  |  |  |  |  | Ascariasis | L | F | 5 | O |  |  |  |
| 132 | *Withania somnifera* (L.) Dun | Solanaceae | Gizawa | S | Headache | R | F/D | 7 | Na |  | 206 |  |
|  |  |  |  |  | Evil eye | R | F/D | 7 | Na |  |  |  |
|  |  |  |  |  | Gonorrhoea | R | F | 1 | O |  |  |  |
|  |  |  |  |  | Syphilis | R | F | 1 | O |  |  |  |
|  |  |  |  |  | Malaise | R | F/D | 7 | Na |  |  |  |
|  |  |  |  |  | Fever | R | F | 7 | Na |  |  |  |
| 133 | *Woodfordia uniflora*  (A. Rich.) Koehne | Lythraceae |  | S | Evil spirit | R | F | 1 | Na |  | 181 |  |
| 134 | *Zehneria scabra* (Linn.f.) Sond. | Cucurbitaceae | Nech Hareg | C | Diarrhoea | L | F | 5 | O |  | 851 |  |
|  |  |  |  |  | Malaise | R,L,St | F | 10 | De |  |  |  |
|  |  |  |  |  | Mumps | R | F | 4 | Au |  |  |  |
|  |  |  |  |  | Fever | R,L,St | F | 10 | De |  |  |  |
|  |  |  |  |  | Taeniasis | L | F | 5 | O |  |  |  |
|  |  |  |  |  | Constipation | R | F/D | 5 | O |  |  |  |
|  |  |  |  |  | Conjunctivitis | L | F | 4 | Op |  |  |  |
| 135 | *Zingiber officinale* Roscoe* | Zingiberaceae | Zingibil | H | Common Cold | Rh | F/D | 1 | O |  | 850 |  |
|  |  |  |  |  | Constipation | Rh | F/D | 1 | O |  |  |  |
|  |  |  |  |  | Coughing | Rh | F/D | 1 | O |  |  |  |
|  |  |  |  |  | Diarrhoea | Rh | F/D | 5 | O |  |  |  |
|  |  |  |  |  | Asthma | Rh | F/D | 1 | O |  |  |  |

Key *Cultivated medicinal plant species ** Endemic species
